# Effects of mulberry twig alkaloids(Sangzhi alkaloids) and metformin on blood glucose fluctuations in combination with premixed insulin-treated patients with type 2 diabetes

**DOI:** 10.3389/fendo.2023.1272112

**Published:** 2023-11-02

**Authors:** Ziyu Meng, Chengye Xu, Haoling Liu, Xinyuan Gao, Xinyu Li, Wenjian Lin, Xuefei Ma, Changwei Yang, Ming Hao, Kangqi Zhao, Yuxin Hu, Yi Wang, Hongyu Kuang

**Affiliations:** Department of Endocrinology, The First Affiliated Hospital of Harbin Medical University, Harbin, China

**Keywords:** mulberry twig alkaloids, metformin, blood glucose fluctuations, premixed insulin, continuous glucose monitors

## Abstract

**Introduction:**

We aimed to evaluated the effect of premixed insulin (Ins), premixed insulin combined with metformin (Ins+Met) or mulberry twig alkaloids(Ins+SZ-A) on blood glucose fluctuations in patients with type 2 diabetes (T2DM) using continuous glucose monitors (CGM).

**Methods:**

Thirty patients with T2DM and poor blood glucose control using drugs were evaluated for eligibility during the screening period. Subsequently, their original hypoglycemic drugs were discontinued during the lead-in period, and after receiving Ins intensive treatment for 2 weeks, they were randomly assigned to receive either Ins, Ins+Met, or Ins+SZ-A treatment for the following 12 weeks. The main efficacy endpoint comprised changes in their CGM indicators changes (mean blood glucose level [MBG], standard deviation of blood glucose [SDBG], mean amplitude of glycemic excursions [MAGE], postprandial glucose excursions [PPGE], the largest amplitude of glycemic excursions [LAGE], mean of daily difference [MODD], time in range between 3.9–10.0 mmol/L [TIR] and area under the curve for each meal [AUCpp]) during the screening, lead-in, and after 12-week treatment period. Changes in glycosylated hemoglobin (HbA1c), fasting blood glucose (FBG), 1-h postprandial blood glucose (1h-PBG), 2-h postprandial blood glucose (2h-PBG), fasting blood lipids and postprandial blood lipids were also measured at baseline and after 12 weeks of treatment

**Results:**

The CGM indicators of the three groups during the lead-in period all showed significant improvements compared to the screening period (P<0.05). Compared with those in the lead-in period, all of the CGM indicators improved in the the Ins+Met and Ins+SZ-A groups after 12 weeks of treatment (P<0.05), except for MODD. After 12-week treatment, compared with the Ins group, Ins+Met and Ins+SZ-A groups showed improved MBG, SDBG, TIR, breakfast AUCpp,lunch AUCpp, HbA1c, FBG, 1h-PBG, fasting blood lipid and postprandial blood lipid indicators (P<0.05). Further, the LAGE, PPGE, MAGE, dinner AUCpp and 2h-PBG levels of the Ins+SZ-A group were significantly lower than those of the Ins+Met and Ins groups (P<0.05).

**Conclusion:**

Our findings highlight the efficacy of combination therapy (Ins+SZ-A or Ins+Met) in improving blood glucose fluctuations, as well as blood glucose and lipid levels. Ins+SZ-A reduces postprandial blood glucose fluctuations more than Ins+Met and Ins groups.

**Trial registration number:**

ISRCTN20835488.

## 1 Introduction

Mulberry twig alkaloids (SZ-A) represent the first original natural hypoglycemic drug to be discovered in China. They are capable of effectively inhibiting α-glycosidase and thus exerting beneficial hypoglycemic effects. One multi-center, randomized, double-blinded, and parallel controlled trial showed that after 24 weeks of treatment with SZ-A, patient levels of glycosylated hemoglobin (HbA1c) decreased by 0.93% from baseline, and their rate of achieving the HbA1c target (HbA1c<7%) was 47.7%—which was equivalent to that of treatment with acarbose for reducing postprandial blood glucose (1). SZ-A is mainly composed of five compounds: 1-deoxynojirimycin, 1,4-dideoxy-1,4-imino-D-arabinitol, fagomine, arginine and polysaccharide. SZ-A can reduce inflammation, regulate gut microbiota, promote glucose-stimulated insulin secretion, promote glucagon-like peptide 1 secretion, and lower weight in obese animals (2, 3). Whether administered by gavage or intraperitoneal injection, SZ-A can both improve non-alcoholic fatty liver disease in obese mice, indicating that the mechanism of its action is independent of its pathway connected to intestinal α- glycosidase. This may be due to the diversity of compounds contained in SZ-A mixtures (4).

When oral hypoglycemic drugs fail to control blood glucose in patients with type 2 diabetes (T2DM), these patients typically begin using insulin (5). T2DM is characterized by insulin resistance, and deficiency. Insulin resistance occurs in the early stages of abnormal glucose metabolism. In the later stages, insulin deficiency is the main factor. Most of these patients eventually require exogenous insulin, and the use of premixed insulin is currently the predominant treatment for this condition in China. One multi-center cross-sectional survey of outpatients in Chinese hospitals showed that approximately 65.6% of patients with diabetes used premixed insulin (6). However, premixed insulin is prone to causing hypoglycemia, which leads to weight gain and poor control of postprandial blood glucose levels (7, 8). A combination of insulin and oral medication can have more comprehensive benefits, such as increasing the effect of peripheral insulin, reducing insulin dosage, reducing the risk of hypoglycemia, improving blood glucose control, and reducing weight gain (9, 10).

Metformin(Met) is the first-line drug for the treatment of T2DM and plays an anti-hyperglycemic role mainly by inhibiting liver glucose through AMPK-dependent or -independent pathways (11–14). The Chinese MERIT study showed that premixed insulin combined with Met resulted in a greater reduction in HbA1c, less insulin consumption, less weight gain, a lower incidence of hypoglycemia, and lower cardiovascular risk than premixed insulin alone (15–17).

Continuous glucose monitors (CGM) can measure glucose concentrations in the subcutaneous interstitial fluid 288 times per day using multiple glucose sensors, allowing them to provide continuous, complete,and reliable glucose measurements with good compliance. To our knowledge, no studies have investigated the effects of SZ-A on blood glucose fluctuations.

Therefore, in this study, CGMs were used to comprehensively evaluate blood glucose changes in patients with T2DM who were treated with premixed insulin combined with SZ-A or Met, compared to other treated with premixed insulin alone. We also considered blood lipid parameters, to provide a basis for exploring the hypoglycemic characteristics of SZ-A and determining the most suitable patients population for this treatment.

## 2 Materials and methods

### 2.1 Study design and participants

This was a 12-week open-label, randomized, parallel-controlled, clinical trial. We enrolled patients with T2DM who had poorly controlled blood glucose levels when using oral hypoglycemic agents, and had been hospitalized in the Department of Endocrinology and Metabolism of the First Affiliated Hospital of Harbin Medical University between June 2022 and March 2023.

The inclusion criteria were patients who: 1) were aged 18–70 years old, regardless of sex; 2) had body mass index(BMI) between 19 kg/m^2^ and 30 kg/m^2^; 3) had been diagnosed with T2DM according to the diagnostic criteria for T2DM formulated by 1999 World Health Organization; 4) were taking oral hypoglycemic agents, who had poor blood glucose control, and had 7% ≤ HbA1c ≤ 10%; 5) were able to understand the procedures and methods of this clinical study, participate voluntarily and sign the informed consent form.

The exclusion criteria were patients who: 1) were allergic or intolerant to α-glucosidase inhibitors, or for whom these drugs had been proven to be ineffective; 2) had severe diabetic complications; 3) had secondary diabetes mellitus, specific types of diabetes, and type 1 diabetes mellitus; 4) had chronic gastrointestinal dysfunction, obvious digestive and absorption disorders, as well as other endocrine diseases, such as hyperthyroidism, Cushing’s syndrome,or acromegaly, etc; 5) had diseases that could be worsened by flatulence (such as Roeheld’s syndrome, severe hernia, intestinal obstruction, following gastrointestinal surgery and intestinal ulcers); 6) had unstable angina pectoris within 6 months prior to the study, had serious heart diseases, or were likely to die during the treatment and follow-up period; 7) had mental and neurological disorders who could not clearly express themselves; 8) were suffering from alcoholism or other substance addictions; 9) were women of childbearing age who were pregnant, lactating, had a positive pregnancy test (urine or blood HCG), intended to become pregnant over the study and follow-up period, or could not take effective contraceptive measures during the study and follow-up period (including measures such as sterilization, intrauterine devices, and oral contraceptives); 10) had participated in clinical trials of other drugs or medical devices in the three months preceding the study’s start date.

This study was approved by the Ethics Review Committee of the First Affiliated Hospital of Harbin Medical University (Harbin, China). All participants provided written informed consent prior to registration. All experiments were conducted in accordance with the principles of the Declaration of Helsinki.

### 2.2 Randomization and treatment

**Figure 1:**
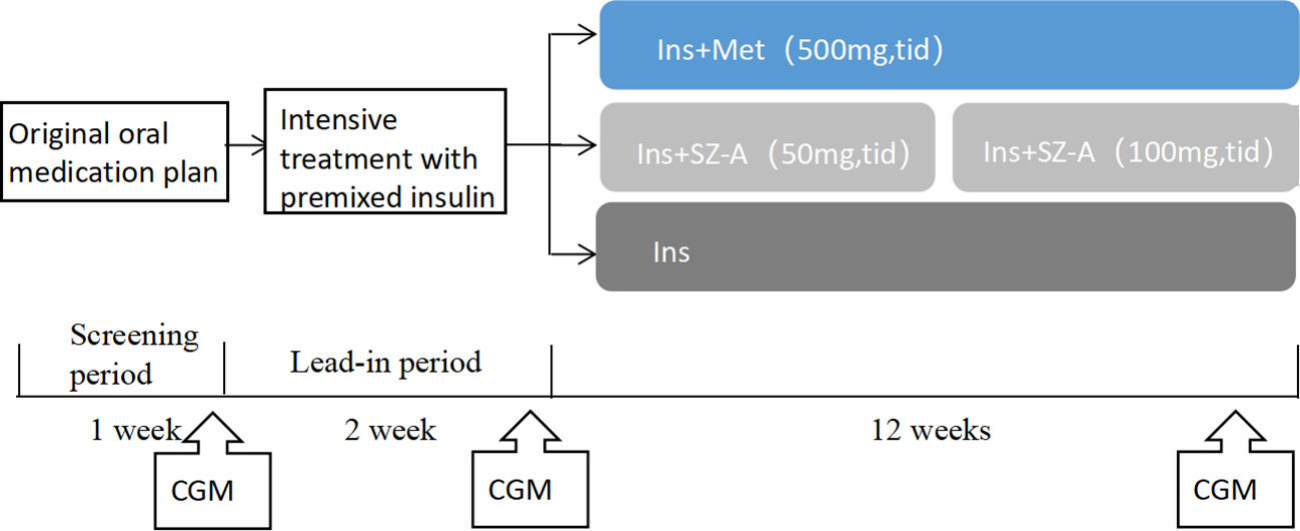
Flowchart of the study.

During the screening period patients used their regular hypoglycemic drugs and evaluations for eligibility to select study subjects were conducted based on the inclusion and exclusion criteria. During the lead-in period, the patients’ regular hypoglycemic drugs were discontinued and a premixed insulin-intensive treatment was administered for 2 weeks to quickly correct hyperglycemia. After 2 weeks of this intensive treatment, the patients were randomly divided into premixed insulin (Ins), premixed insulin plus SZ-A (Ins+SZ-A) and premixed insulin plus Met (Ins+MET) groups for the following 12 weeks (**Figure 1**). Premixed insulin was defined as a mixed protamine-zinc recombinant human insulin lispro injection(50 R; Lilly France, IN, USA).

The goal in terms of fasting blood glucose control for this study was 4.4–7.0 mmol/L. The initial premixed insulin dose was 0.4–0.5 IU/kg, after which, it was adjusted as needed, according to plasma glucose values obtained through self-monitoring. The initial dose of SZ-A (Beijing Guokaihua Intellectual Property Agency Co., LTD, Beijing, China) was 50mg, administered three times per day, at mealtimes. After four weeks, this dose was increased to 100mg three times per day at mealtimes. The Met dose (Sino-American Shanghai Squibb Pharmaceuticals Co., Ltd, Shanghai, China) was 500mg three times per day at mealtimes, throughout the entire observation period. The first 6 weeks of the treatment phase comprised the insulin dose titration period. Patients were required to self-monitor their blood glucose levels, and a trained doctor adjust their insulin doses based on the results obtained. The following 6 weeks comprised the steady dose period. The patients received diabetes education during this phase, in order to promote reasonable dietary control and proper exercise over the rest of the study period.

### 2.3 Anthropometric indicators

The general patients information collected included sex, age,diabetes history, height, weight, BMI, waist circumference, hip circumference, and insulin dose. Fasting blood glucose (FBG), HbA1C, C-peptide, cholesterol (CHOL), triglyceride (TG), total cholesterol (TC), high-density lipoprotein cholesterol (HDL-C), and low-density lipoprotein cholesterol (LDL-C) levels were measured during the screening period and after 12 weeks of treatment. An automatic biochemical analyzer assessed hypoglycemia, adverse events, alanine aminotransferase (ALT), aspartate transaminase (AST), creatinine (Cr), and uric acid (UA).

The insulin resistance index was assessed for each patients using modified homeostasis model, and expressed as [HOMA-IR(CP)] = 1.5 + FPG (mmol/L) × fasting C-peptide (pmol/L)/2,800 (18).

The oral glucose tolerance tests (OGTT) experiment was conducted during the lead-in period, where participants received 75 g of orally-administered glucose in the morning after fasting for 10 to 12h. Blood samples were collected at 0, 60, and 120 min afterward, to measure plasma glucose concentrations.

After 12 weeks of treatment, the participants fasted overnight, were treated with their corresponding hypoglycemic drugs (Ins, Ins+Met, or Ins+SZ-A), and consumed 70 g of instant noodles (500 kcal, 70 g of carbohydrates) within 15 min (19). Blood samples were collected before the test meal, and at 60 and 120 min afterward, to measure FBG, 1-hour postprandial blood glucose (1h-PBG), 2-hour postprandial blood glucose (2h-PBG), fasting blood lipids, 1-hour postprandial blood lipids, and 2-hour postprandial blood lipids. The area under the PBG curve (BG AUC) and the area under the curves for the other blood lipid indicators were then calculated.

### 2.4 CGM parameters

Subcutaneous interstitial glucose monitoring was conducted using a CGM system (Medtronic, Inc,Minnesota,USA) during the screening, lead-in, and after 12-week treatment period, for three consecutive days. The study parameters included mean blood glucose level (MBG), standard deviation of blood glucose (SDBG), mean amplitude of glycemic excursions (MAGE), postprandial glucose excursions (PPGE), largest amplitude of glycemic excursions (LAGE), mean of daily difference (MODD), and time-in-range between 3.9 and 10.0 mmol/L (TIR). The area under the curve (AUCpp) was calculated within 4 h of the start of each meal.

### 2.5 End point

The primary endpoints included changes in MBG, SDBG, MAGE, PPGE, LAGE, MODD, TIR, breakfast AUCpp, lunch AUCpp, and dinner AUCpp readings during the screening period, lead-in period, and after 12 weeks of treatment.

The secondary endpoints were changes in HbA1c, FBG, 1h-PBG, 2h-PBG, fasting blood lipids, postprandial blood lipids at baseline and after 12 weeks of treatment.

### 2.6 Statistical method

All statistical analyses were performed using SPSS 25.0. For normally-distributed numerical variables, one-way analysis of variance (ANOVA) was used to compare differences between groups, and paired Student’s t-tests were used before and after treatment to assess differences in intra-group outcome measures. For non-normally distributed variables, the Kruskal–Wallis test was used to compare intergroup differences, and the Wilcoxon signed-rank test was used to assess differences in intra-group outcome measures before and after the interventions. The Bonferroni method was used for *post-hoc* multiple comparisons. For categorical variables, Fisher’s exact test was used for comparisons between groups. The α-level was set at 5%, and the significance level at 95%. Statistical significance was set at P < 0.05.

## 3 Results

### 3.1 Baseline patient characteristics

We included 30 patients with T2DM for whom blood glucose levels could not be successfully controlled. Of them, 10 were assigned to each of the INS, INS+Met, and INS+SZ-A groups. No significant differences were observed in the general characteristics (such as age, sex ratio, weight, BMI, waist circumference, waist-to-hip ratio, and duration of diabetes) and efficacy and safety indicators (including CHOL, TG, HDL,LDL, FBG, HbA1c, HOMA-IR (CP), ALT, AST, Cr, UA, OGTT AUC (h·mmol/L), and insulin dose)between the three groups (P >0.05; **Table 1**).

**Table 1:** Baseline data of three groups.

	Ins+Met	Ins+SZ-A	Ins	P
Age (year)	49.00 ± 12.86	48.40 ± 13.76	51.40 ± 9.06	0.842
Sex (Female/Male)				0.500
Female	7 (70.00%)	8 (80.00%)	5 (50.00%)	
Male	3 (30.00%)	2 (20.00%)	5 (50.00%)	
Body weight (kg)	73.85 ± 8.79	64.55 ± 8.78	67.95 ± 8.48	0.070
BMI (kg/m^2^)	25.18 ± 2.61	22.95 ± 3.03	24.56 ± 2.00	0.154
Waist circumference (cm)	89.75 ± 3.39	86.40 ± 7.62	89.40 ± 6.33	0.407
waist-to-hip ratio	0.93 ± 0.05	0.94 ± 0.07	0.96 ± 0.06	0.554
diabetic duration (year)	8.10 ± 5.45	8.60 ± 4.99	7.80 ± 5.12	0.941
HbA1c (%)	9.65 (7.38,9.80)	9.65 (8.68,9.80)	9.70 (9.38,9.80)	0.531
FPG (mmol/L)	9.31 ± 1.73	9.79 ± 3.32	10.85 ± 2.79	0.438
Insulin dose (U)	29.00 (24.00.34.50)	32.00 (20.50,33.25)	36.00 (28.00,36.50)	0.093
HOMA-IR (CP)	3.35 ± 0.79	3.15 ± 0.78	3.62 ± 0.86	0.436
OGTT AUC (h·mmol/L)	29.00 ± 5.92	28.48 ± 2.72	28.65 ± 3.25	0.961
CHOL (mmol/L)	4.65 ± 0.86	4.99 ± 1.52	4.68 ± 0.98	0.764
TG (mmol/L)	2.33 (0.98.4.10)	1.97 (1.34,2.74)	2.18 (1.40,3.78)	0.882
HDL (mmol/L)	0.89 (0.69,1.02)	1.07 (1.00,1.32)	0.90 (0.73,1.07)	0.070
LDL (mmol/L)	2.67 ± 0.72	3.12 ± 1.28	2.81 ± 0.72	0.564
AST (U/L)	15.93 ± 3.23	16.64 ± 4.25	15.82 ± 4.47	0.885
ALT (U/L)	21.72 ± 7.36	20.10 ± 7.62	20.96 ± 10.66	0.917
Cr (umol/L)	52.92 ± 7.34	51.84 ± 8.85	47.70 ± 11.48	0.433
UA (umol/L)	306.83 ± 68.55	306.94 ± 77.27	303.81 ± 59.41	0.933

All P>0.05, Fisher’s precision probability test, one-way ANOVA, Kruskal-Wallis H test or Fisher’s precision probability test were performed among the three groups.

### 3.2 General conditions at baseline and after the 12-week treatment

Compared those during screening period, HbA1c and FBG in the three groups were significantly improved after 12 weeks of treatment (P<0.05), but weight changes were not statistically significant (P>0.05). Compared with those during the lead-in period, the insulin doses in the Ins+Met and Ins+SZ-A groups decreased after 12 weeks of treatment (P<0.05). After 12-week treatment, the HbA1c, FBG and insulin dosages of Ins+Met and Ins+SZ-A groups were lower than those of the Ins group (P<0.05), and there was no statistically significant difference in weight between the three groups (P>0.05; **Table 2**).

**Table 2:** General conditions at baseline and after 12-week treatment.

	Ins+Met		Ins+SZ-A		Ins	
	Baseline	After 12 weeks	Baseline	After 12 weeks	Baseline	After 12 weeks
HbA1c (%)	9.65 (7.38,9.80)	6.70 (6.48,7.35)^*#^	9.65 (8.68,9.80)	6.65 (6.25,7.60)^*&^	9.70 (9.38,9.80)	8.30 (7.40,8.75)^*^
FPG (mmol/L)	9.31 ± 1.73	6.90 ± 0.97^*#^	9.79 ± 3.32	6.83 ± 1.20^*&^	10.85 ± 2.79	7.93 ± 0.94^*^
Body weight (kg)	73.85 ± 8.79	73.05 ± 8.02	64.55 ± 8.78	64.15 ± 8.58	67.95 ± 8.48	68.45 ± 8.28
Insulin dose (U)	29.00 (24.00.34.50)	19.00 (15.5,28.50)^*#^	32.00 (20.50,33.25)	21.00 (18.50,30.50)^*&^	36.00 (28.00,36.50)	35.00 (31.00,38.00)
CHOL (mmol/L)	4.65 ± 0.86	4.69 ± 0.94	4.99 ± 1.52	4.31 ± 1.02	4.68 ± 0.98	4.38 ± 0.98
TG (mmol/L)	2.33 (0.98.4.10)	1.38 (0.88,1.64)^*#^	1.97 (1.34,2.74)	0.83 (0.64,1.01)^*&^	2.18 (1.40,3.78)	2.07 (1.66,3.53)
HDL (mmol/L)	0.89 (0.69,1.02)	1.35 (1.10,1.59)^*#^	1.07 (1.00,1.32)	1.23 (1.11,1.33)^*&^	0.90 (0.73,1.07)	0.89 (0.76,0.95)
LDL (mmol/L)	2.67 ± 0.72	2.64 ± 0.68	3.12 ± 1.28	2.37 ± 0.76^*^	2.81 ± 0.72	2.52 ± 0.82^*^

*represents P < 0.05 (comparison between baseline and after 12-week treatment in each group).

^#^represents P < 0.05 (comparison between Ins+Met and Ins groups after 12-week treatment).

^&^represents P < 0.05 (comparison between Ins+SZ-A and Ins groups after 12-week treatment).

Compared with those in the screening period, the TG and HDL indicators of the Ins+Met group (P<0.05), the TG, HDL and LDL levels of the Ins+SZ-A group (P<0.05), and the LDL levels of the Ins group (P<0.05) all improved after the 12 weeks of treatment, there were no significant differences in the other indicators (P<0.05). After 12 weeks treatment, the improvement in TG and HDL levels seen in the Ins+Met and Ins+SZ-A groups was greater than those in the Ins group (P<0.05). There were no significant differences in the other indicators (P>0.05; **Table 2**).

### 3.3 Fasting and postprandial blood glucose and lipid levels after 12 weeks of treatment

**Figure 2:**
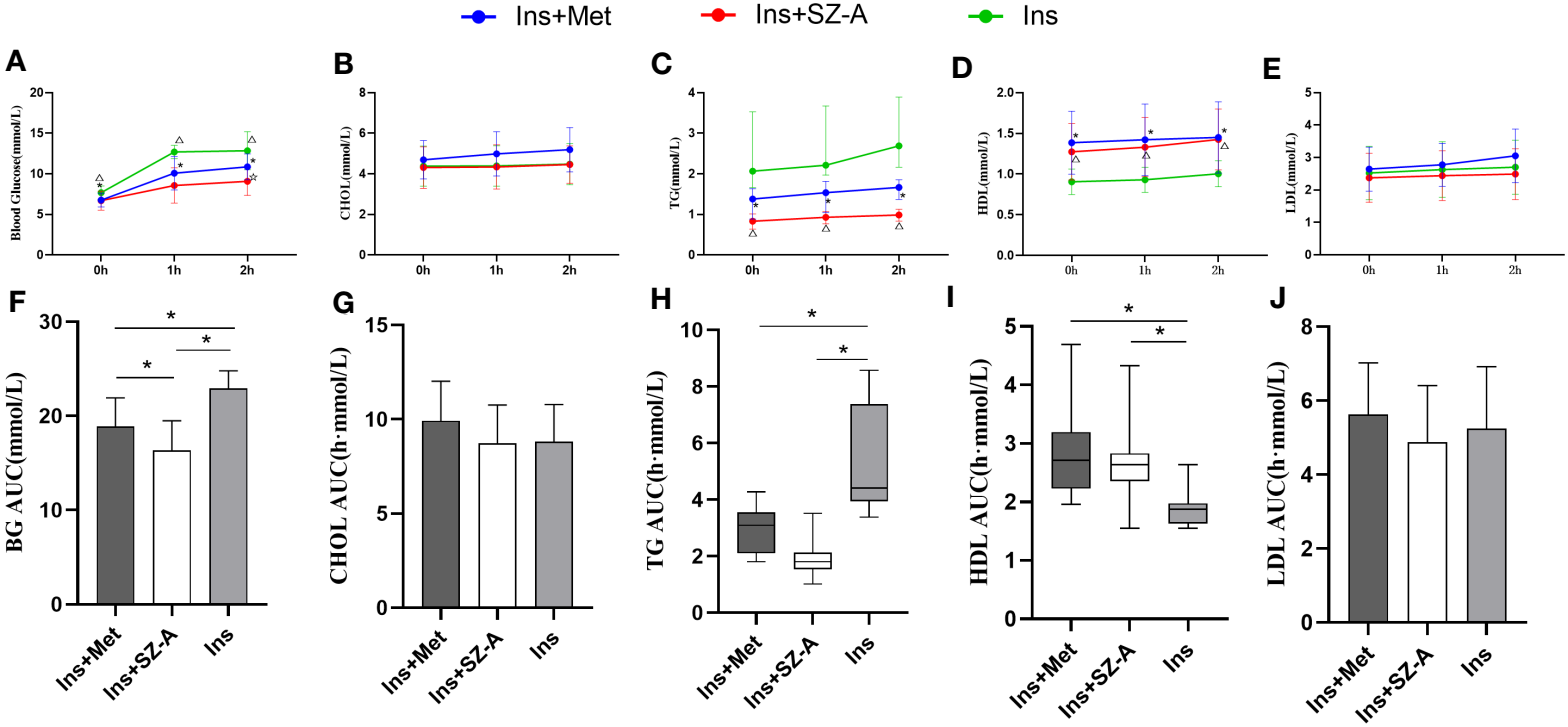
Fasting and Postprandial Blood Glucose and Lipid Levels After 12 Weeks of Treatment **(A)** changes in fasting and postprandial blood glucose levels in three groups. **(B)** changes in fasting and postprandial CHOL levels in three groups. **(C)** changes in fasting and postprandial TG levels in three groups. **(D)** changes in fasting and postprandial HDL levels in three groups. **(E)** changes in fasting and postprandial LDL levels in three groups. *P<0.05 Ins vs Ins+Met, △P<0.05 Ins vs Ins+SZ-A, ☆P<0.05 Ins+Met vs Ins+SZ-A, BG AUC **(F)**,CHOL AUC **(G)**, TG AUC **(H)**,HDL AUC **(I)**,LDL AUC **(J)** levels among the groups. *P<0.05.

After 12 weeks of treatment, FBG and 1h-PBG levels were lower in the Ins+Met and Ins+SZ-A groups than in the Ins group (P<0.05;**Figure 2A**). The 2h-PBG and BG AUC were also lower in the Ins+SZ-A group than in the Ins+Met and Ins groups (P<0.05; **Figure 2F**).

After 12 weeks of treatment, the fasting TG, 1-hour postprandial TG, 2-hour postprandial TG, and TG AUC indicators were lower in the Ins+Met and Ins+SZ-A groups than in the Ins group (P<0.05; **Figures 2C, H**). The fasting HDL, 1-hour postprandial HDL, 2-hour postprandial HDL,and HDL AUC were also higher in the Ins+Met and Ins+SZ-A groups than in the Ins group (P<0.05; **Figures 2D, I**). There were no statistically significant differences observed in terms of the other indicators (P>0.05; **Figures 2B, E, G, J**).

### 3.4 Continuous glucose monitoring results

**Figure 3:**
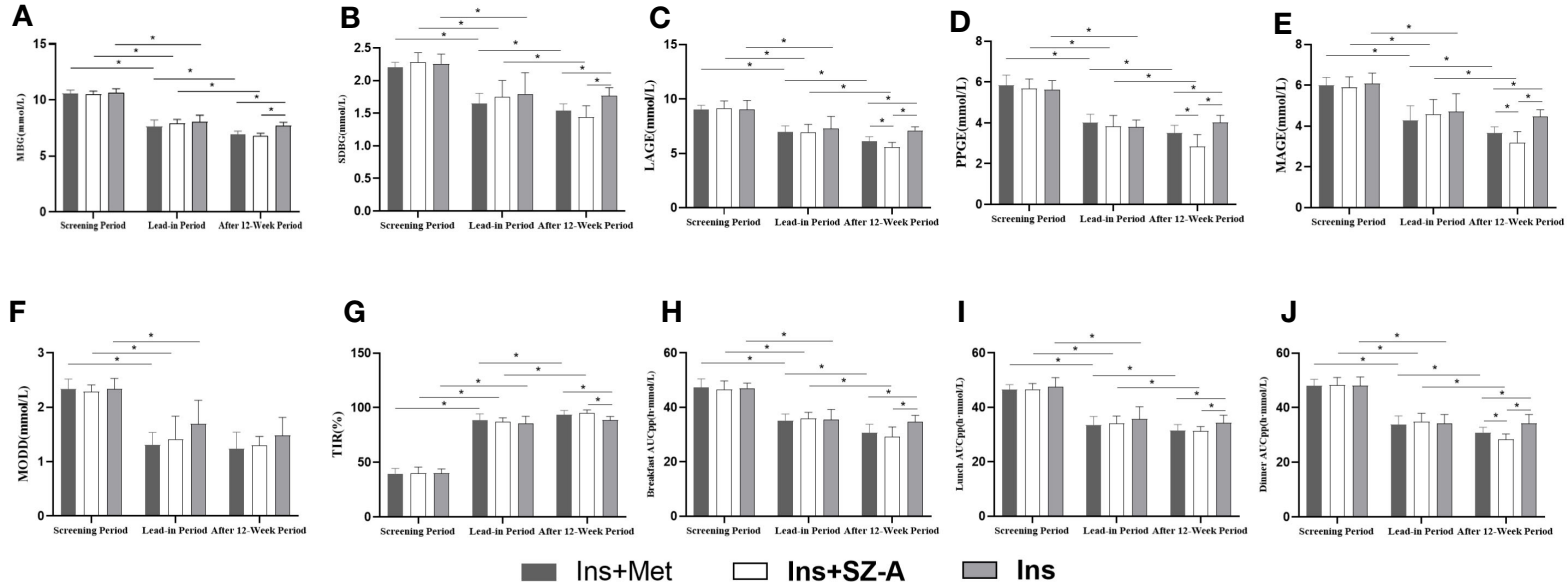
CGM results Changes in MBG **(A)**, SDBG **(B)**, LAGE **(C)**, PPGE **(D)**, MAGE **(E)**, MODD **(F)**, TIR **(G)**, breakfast AUCpp **(H)**, lunch AUCpp **(I)**, and dinner AUCpp **(J)** at different stages among the three groups. * P<0.05.

No statistically significant differences were noted in terms of CGM indicators such as MBG, SDBG, LAGE, PPGE, MAGE, MODD, TIR, breakfast AUCpp, lunch AUCpp, and dinner AUCpp among the three groups during the screening and lead-in period (P>0.05). Compared to the screening period, the MBG, SDBG, LAGE, PPGE, MAGE, MODD, TIR, breakfast AUCpp, lunch AUCpp, and dinner AUCpp indicators of all three groups showed significant improvements during the lead-in period (P<0.05; **Table 3**; **Figure 3**).

**Table 3:** CGM results.

	Ins+Met			Ins+SZ-A			Ins		
	Screeing period	Lead-in period	After 12 weeks	Screeing period	Lead-in period	After 12 weeks	Screeing period	Lead-in period	After 12 weeks
MBG(mmol/L)	10.59±0.30	7.64±0.57*	6.91±0.32#	10.49±0.29	7.91±0.35*	6.78±0.25#	10.65±0.36	8.03±0.62*	7.71±0.31
SDBG(mmol/L)	2.20±0.07	1.65±0.15*	1.54±0.11#	2.28±0.15	1.75±0.25*	1.44±0.17#	2.26±0.15	1.80±0.32*	1.77±0.12
LAGE(mmol/L)	9.03±0.40	6.98±0.55*	6.11±0.44#	9.17±0.65	6.95±0.71*	5.59±0.41#	9.04±0.83	7.28±1.13*	7.10±0.34
PPGE(mmol/L)	5.84±0.50	4.03±0.40*	3.52±0.37#	5.69±0.46	3.83±0.54*	2.83±0.60#	5.64±0.43	3.80±0.34*	4.02±0.35
MAGE(mmol/L)	6.00±0.39	4.29±0.71*	3.66±0.31#	5.90±0.50	4.58±0.72*	3.19±0.54#	6.08±0.50	4.71±0.87*	4.46±0.35
MODD(mmol/L)	2.33±0.19	1.31±0.22*	1.24±0.30	2.28±0.13	1.42±0.42*	1.30±0.17	2.33±0.20	1.70±0.44*	1.48±0.33
TIR(%)	39.78±4.80	88.44±5.93*	93.65±3.75#	40.24±5.51	86.96±3.67*	94.98±2.99#	40.19±3.84	85.45±6.58*	88.62±3.37
Breakfast AUCpp(h·mmol/L)	47.34±3.14	35.18±2.39*	30.71±3.06#	46.54±3.20	35.87±2.29*	29.25±3.52#	47.06±1.88	35.48±3.76*	34.69±2.39
Lunch AUCpp(h·mmol/L)	46.57±1.82	33.59±3.11*	31.45±2.22#	46.66±2.14	34.04±2.78*	31.23±1.68#	47.55±3.37	35.73±4.49*	34.28±2.85
Dinner AUCpp(h·mmol/L)	48.05±2.38	33.89±3.07*	30.91±1.94#	48.22±2.86	34.85±3.08*	28.47±1.90#	48.04±3.22	34.16±3.29*	34.21±3.27

*represents P < 0.05(comparison between screening and lead-in period for each group,matched-samples Student’s t- test).

#represents P < 0.05(comparison between lead-in and after 12-week treatment period for each group,matched-samples Student’s t- test).

Compared with those during the lead-in period, the Ins+Met and Ins+SZ-A groups showed significant improvements in terms of MBG, SDBG, LAGE, PPGE, MAGE, TIR, breakfast AUCpp, lunch AUCpp, and dinner AUCpp after the 12-week treatments (P<0.05); there was no statistically significant difference observed in MODD (P>0.05). None of these indicators improved significantly in the Ins group (P>0.05). After 12 weeks of treatment,the MBG, SDBG, TIR,breakfast AUCpp, and lunch AUCpp indicators of the Ins+Met and Ins+SZ-A groups showed significantly greater than those of the Ins group (P<0.05).In addition, the LAGE, PPGE, MAGE, and dinner AUCpp levels of the Ins+SZ-A group were lower than those of the Ins+Met nd Ins groups (P<0.05). However,there were no statistically significant differences in MODD among the three groups (P>0.05; **Table 3**; **Figure 3**).

### 3.5 Adverse reactions

After 12 weeks of treatment, there were no statistically significant changes in ALT, AST, Cr, and UA in any of the groups (P>0.05). The three treatment regimens did not show any severe hypoglycemic reactions during any of the study phases. There were no significant differences in the hypoglycemic responses of the three groups during the screening, lead-in, and after 12-week treatments periods (P>0.05). One case of abdominal distension and one case of diarrhea occurred in the Ins+Met group, and the Ins+SZ-A group experienced one case of abdominal distension and no cases of diarrhea. The patients who experienced these events received appropriate medications administered during mealtimes, starting with low doses that were then gradually increased until the adverse reactions were ameliorated, without altering their main treatment regimens.

## 4 Discussion

It is important to reach the target levels for blood glucose and HbA1c when treating patients with T2DM. Good control over blood glucose fluctuations is also important. Patients with diabetes who have similar HbA1c levels may have different blood glucose stabilities, and large blood glucose fluctuations may be associated with a greater risk of diabetic complications (20). A higher TIR has been linked to reduced risks of albuminuria, retinopathy, cardiovascular disease mortality, all-cause mortality, and abnormal carotid intima-media thickness. Peripheral neuropathy is associated with SDBG and MAGE; therefore, strengthening the management of blood glucose fluctuations plays a key role in preventing macrovascular and microvascular complications related to diabetes (21, 22). Hyperlipidemia also increases the risk of atherosclerotic cardiovascular disease in patients with diabetes (23). Postprandial hyperlipidemia is an important risk factor, particularly in patients who have both metabolic syndrome and diabetes (24). Studies have shown that SZ-A can improve fasting blood glucose and lipids levels, but there are currently no data on blood glucose fluctuations and postprandial blood lipids in patients taking SZ-A (1, 4).

In this study, the three treatment regimens all improved the patients’ blood glucose levels, and the results in the Ins+SZ-A group were superior to those in the Ins+Met and Ins groups in terms of improving postprandial blood glucose fluctuations. All three treatment regimens reduced fasting blood lipids, but the Ins+Met and Ins+SZ-A treatments also improved the patients’ postprandial blood lipid indicators.

HbA1c, which reflects long-term blood glucose control, has become the gold standard for evaluating blood glucose control and guiding clinical decisions regarding the management of diabetes. Compared to during the screening period, the HbA1c and FBG levels in all three groups decreased following the 12-week treatment period. After 12 weeks treatment, the HbA1c, FBG, and administered insulin doses in the Ins+Met and Ins+SZ-A groups were lower than those in the Ins group. The insulin doses in the Ins+Met and Ins+SZ-A groups during the lead-in period were 29.00 (24.00, 34.5) and 32.00 (20.50, 33.25), respectively. Following the 12-week treatment period, these doses decreased to 19.00 (15.50, 28.50) and 21.00 (18.50, 30.50), respectively. Although the absolute values of insulin doses in the Ins+SZ-A group were greater than those in the Ins+Met group, both during the lead-in period and after 12 weeks of treatment, this difference was not statistically significant. Therefore, we believe that drugs, rather than insulin, decrease hypoglycemia and hyperlipidemia.

TIR is a key CGM indicator that describes short-term blood glucose control and quantifies the time within the target range (25). Research has shown a correlation between HbA1c and TIR levels, with a 10% change in TIR being equated to a 0.8% change in HbA1C (26). In this study, the TIR levels were higher during the lead-in period for all three groups, compared to during the screening period. However, when compared to the lead-in period, the TIR of the Ins+Met and Ins+SZ-A groups were found to be significantly improved after the 12-week treatment period. There was no significant difference found in terms of this indicator in the Ins group. The TIR of the Ins+Met and Ins+SZ-A groups were both higher than those of the Ins group after 12 weeks of treatment. This indicates that insulin combined with oral medication can improve short-term blood glucose control more effectively than insulin alone.

MBG reflects the average blood glucose level, whereas SDBG reflects the magnitude of overall deviations in glucose levels from the average (27, 28). In our study, MBG and SDBG levels of all of the groups were lower during the lead-in period than during the screening one. However, when compared to the lead-in period, the Ins+Met and Ins+SZ-A groups showed significant decreases in MBG and SDBG levels after 12 weeks treatment. And after 12 weeks treatment,the MBG and SDBG levels in the Ins+Met and Ins+SZ-A groups were lower than those in the Ins group, indicating that the blood glucose levels in the Ins+Met and Ins+SZ-A groups were closer to normal than those in the Ins group.

LAGE is the difference between the maximum and minimum daily glucose levels, and may be an independent predictor of nocturnal asymptomatic hypoglycemia in patients with T2DM. LAGE measurements of >3.48 mmol/L can be used as an early warning sign of nocturnal asymptomatic hypoglycemia (29). MAGE is the average value obtained by removing all blood glucose fluctuations with an amplitude of <1SD, and is the gold standard for evaluating blood glucose fluctuations within a single day. A MAGE measurements of <3.9 mmol/L is recommended as the normal reference range for blood glucose fluctuations in Chinese adults (28). In our study, the LAGE and MAGE levels of all of the groups were lower during the lead-in period than during the screening period. Compared to the lead-in period, the LAGE and MAGE measurements of the Ins+Met and Ins+SZ-A groups were significantly reduced after 12-week treatment. After the 12-week treatment, the LAGE and MAGE indicators of the Ins+SZ-A group were lower than those of the Ins+Met and Ins groups, indicating that, compared to the Ins+SZ-A and Ins groups, Ins+SZ-A is able to better stabilize within-day blood glucose fluctuations.

MODD reflects day-to-day blood glucose excursions, which are the differences between blood glucose values measured at the same time point on two consecutive days. The MODDs of all three groups in our study cohort were lower during the lead-in period than during the screening period. There were no statistically significant differences observed in terms of MODDs among the three groups during the lead-in period or after 12-week treatment period.

Postprandial blood glucose control is crucial for achieving overall blood glucose control, with postprandial hyperglycemia being the main factor that leads to general hyperglycemia (30). Postprandial hyperglycemia is generally believed to be a predictor of cardiovascular diseases and microvascular complications (31, 32). Therefore, it is necessary to consider postprandial glucose control as an important strategy in the comprehensive treatment plan for patients with diabetes. The PPGEs of all three groups in our study were lower during in the lead-in period than in the screening one. Compared with those during the lead-in period, the Ins+Met and Ins+SZ-A groups showed significant improvements in PPGE measurements after the 12-week treatment period, whereas the Ins group showed no significant changes in PPGE. The PPGE and 2h-PBG levels of the Ins+SZ-A group were lower than those of the Ins+Met and Ins groups after 12 weeks of treatment. This indicates that Ins+SZ-A may improve postprandial blood glucose fluctuations more effectively compared with the Ins+Met and Ins groups.

After the 12 weeks of treatment, the dinner AUCpp of the Ins+SZ-A group was lower than that of the Ins+Met and Ins groups, indicating that the Ins+SZ-A group experienced a more significant improvement in post-dinner blood glucose levels, which may be partially due to the cumulative effect of α-glucosidase inhibitors (33). Although no human data available, it has been reported that the turnover time of disaccharidase in rats is 11.5 h. Thus, when SZ-A is taken at every meal, its cumulative effects are most observable at dinnertime (34). This may also be due to differences in the nutritional composition of each meal, and the higher carbohydrate content generally found in Chinese dinners. Thus, SZ-A may be more effective at controlling postprandial hyperglycemia in the Chinese population.

Animal experiments have shown that SZ-A significantly reduces liver weight, liver triglycerides, and total cholesterol levels. However, there is still no data regarding the effects of SZ-A on postprandial blood lipids (4). In our experiment, we verified that SZ-A was able to effectively improve blood lipid levels. After 12 weeks of treatment, HDL increased and both TG and LDL decreased in the Ins+SZ-A group, whereas HDL increased and TG decreased in the Ins+Met group. Furthermore, the Ins+SZ-A and Ins+SZ-A groups showed improved postprandial blood lipid levels. Compared with the Ins group, the Ins+SZ-A and Ins+SZ-A groups showed better-corrected postprandial TG and HDL levels.

Gastrointestinal side effects are one of the limitations to the clinical application of α-glycosidase inhibitors and Met. These may include flatulence, abdominal distension, diarrhea, abdominal pain, and other symptoms. In our experiment, the incidence of GDs in the Ins+SZ-A group was very low. *In vitro* experiments have shown that SZ-A exerts a strongly inhibitory effect on maltase (IC50 = 0.06 μG/mL) and sucrase (IC50 = 0.03 μG/mL).With regard to α-amylase, however, SZ-A had no inhibitory activity at 100 μg/mL (35). Therefore, SZ-A selectively inhibits disaccharidases in order to reduce postprandial hyperglycemia. These findings may partially explain the mechanism underlying the low incidence of GDs observed in the Ins+SZ-A group in our research.

To the best of our knowledge, this is the first research to evaluate the effects of SZ-A or Met combined with premixed insulin on blood glucose fluctuations in patients with T2DM. The combination of SZ-A or Met with premixed insulin not only improved blood glucose control, but also reduced blood glucose fluctuations and blood lipid indicators in our cohort of patients with T2DM whose blood glucose levels could not be controlled through the use of oral medications. SZ-A combined with premixed insulin proved to be better for reducing postprandial blood glucose fluctuations than Met combined with premixed insulin and premixed insulin alone. However, this study also had some limitations worth noting. This was a single-center study with a relatively small number of patients and a study period of only 3 months. Thus, it would be best to extend the treatment period to 6 months or 1 year. Further multi-center studies with larger sample sizes are also warranted to evaluate the safety and efficacy of long-term treatment with SZ-A.

The results of this study suggest that the combination of SZ-A and Met with premixed insulin is a potential treatment option for patients with T2DM whose blood glucose levels cannot be adequately controlled by oral medications, and SZ-A combined with premixed insulin may be more suitable for Chinese patients who consume higher levels of carbohydrates. Further prospective studies with more patients over longer periods are required to verify this hypothesis.

## Data availability statement

The original contributions presented in the study are included in the article/supplementary material. Further inquiries can be directed to the corresponding author.

## Ethics statement

The studies involving humans were approved by Ethics Review Committee of the First Affiliated Hospital of Harbin Medical University. The studies were conducted in accordance with the local legislation and institutional requirements. The participants provided their written informed consent to participate in this study. Written informed consent was obtained from the individual(s) for the publication of any potentially identifiable images or data included in this article.

## Author contributions

ZM: Writing – original draft. CX: Writing – original draft. HL: Writing – original draft. XG: Writing – original draft. XL: Writing – original draft. WL: Writing – original draft. XM: Writing – original draft. CY: Writing – original draft. MH: Writing – original draft. KZ: Writing – original draft. YH: Writing – original draft. YW: Writing – original draft. HK: Writing – review & editing.
